# Predicting Chemical End-of-Life Scenarios Using Structure-Based Classification Models

**DOI:** 10.1021/acssuschemeng.2c05662

**Published:** 2023-02-24

**Authors:** Jose D. Hernandez-Betancur, Gerardo J. Ruiz-Mercado, Mariano Martin

**Affiliations:** † Department of Chemical Engineering, University of Salamanca, Salamanca 37008, Spain; ‡ Office of Research & Development, US Environmental Protection Agency, Cincinnati, Ohio 45268, United States; § Chemical Engineering Graduate Program, Universidad del Atlántico, Puerto Colombia 080007, Colombia

**Keywords:** end-of-life, exposure scenarios, machine learning, QSAR modeling, classification model, industrial chemicals

## Abstract

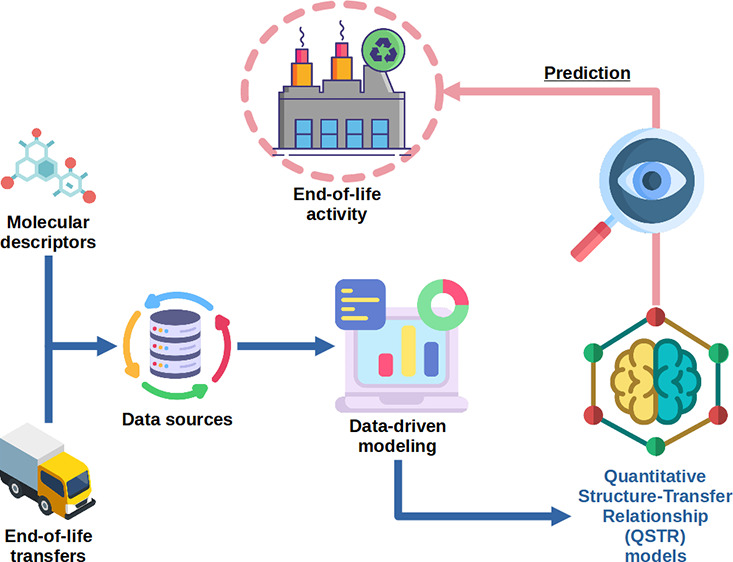

Analyzing chemicals and their effects on the environment from a life cycle viewpoint can produce a thorough analysis that takes end-of-life (EoL) activities into account. Chemical risk assessment, predicting environmental discharges, and finding EoL paths and exposure scenarios all depend on chemical flow data availability. However, it is challenging to gain access to such data and systematically determine EoL activities and potential chemical exposure scenarios. As a result, this work creates quantitative structure-transfer relationship (QSTR) models for aiding environmental managment decision-making based on chemical structure-based machine learning (ML) models to predict potential industrial EoL activities, chemical flow allocation, environmental releases, and exposure routes. Further multi-label classification methods may improve the predictability of QSTR models according to the ML experiment tracking. The developed QSTR models will assist stakeholders in predicting and comprehending potential EoL management activities and recycling loops, enabling environmental decision-making and EoL exposure assessment for new or existing chemicals in the global marketplace.

## Abstract 2

This contribution aims at chemical end-of-life scenarios identification, thereby understanding the complete chemical life cycle and assessing environmental and human health impacts.

## Introduction

Chemicals are present in most commercial products and play an important role in the everyday life of people around the world. Some of them need to be properly managed to avoid harmful effects on human health and the environment.^1^ Chemical exposure assessment looks to determine those events or exposure scenarios that may result in adverse environmental and human health outcomes, either to understand their causes or to prevent them.^2^ Collecting comprehensive data about the conditions of use of chemicals is an important step in developing an exposure scenario and performing chemical life cycle risk assessment.^3,4^ The conditions of use are the scenarios in which a chemical substance is intended, known, or reasonably anticipated to be produced, processed (including recycling), distributed commercially, utilized, or disposed of (including treatments).^5^ The unavailability of data on conditions of use, material flows, and activities as well challenges and time collecting such data are currently hindrances to chemical exposure and risk evaluation studies.^6^ These issues could result, for example, in businesses inappropriately assessing alternative chemicals to reduce the impacts caused by chemicals of concern^7^ or determining whether their products satisfy safety standards before going into the market.^8^ In addition, the increasing number of chemicals circulating in the market means that governments face challenges in identifying all chemical exposure scenarios with unreasonable risks, thereby not ruling quickly with conditions and restrictions for their safer use.^9^ Data collection is even more uncertain at the EoL stage due to the lack of knowledge required to track the chemical flows through the EoL management chain.^10^

Material flow analysis can help map EoL flow movements and EoL activities,^11^ offering the transfer factors for all EoL activities when multiple entities are involved. Then, those transfer factors provided by the material flow analysis can be used either for life cycle impact assessment or exposure scenario identification.^12,13^ In fact, material flow analysis has been used by practitioners to obtain chemical flow inventories for a large number of chemicals.^14−16^ For instance, as part of the program run by the Organization for Economic Cooperation and Development (OECD)’s Working Group on Environmental Information and Outlooks, a material flow analysis was used to determine environmental releases and exposure routes of mercury and quantitatively assess risk to human health endpoints like digestive and immune system.^17^ Hernandez-Betancur et al.^18^ developed a data engineering framework that uses facility-level information from US siloed and publicly accessible databases to perform chemical flow analysis (CFA) of EoL chemical flows transferred to off-site facilities for further EoL management. Hernandez-Betancur et al.^19^ used data at the pollution abatement unit technology level to perform CFA and allocate chemical flows downstream of the pollution abatement units. Hernandez-Betancur et al.^20^ combined the above two frameworks^18,19^ and extended them beyond the EoL management chain boundaries to identify and track recycled chemical flows. Using a Markov random field model, Hernandez-Betancur et al.^20^ outlined the relationship between the entities through the EoL management chain and recycling loops. Due to the legal, economic, market, and policy implications, the behavior of the EoL management chain may vary from country to country and year to year.^21^ In addition, to create a database called PRTR_transfers that contains global inventory data of EoL chemical off-site transfers reported by industrial facilities and businesses, Hernandez-Betancur et al.^22^ expanded the EoL data engineering framework suggested by Hernandez-Betancur et al.^18^ by incorporating data from siloed (i.e., isolated) and publicly accessible international database systems. Thus, the data engineering frameworks can supply the EoL CFA and life cycle inventory (LCI) data of existing chemicals reported in regulatory primary data sources.

Machine learning (ML) has been widely used by researchers and practitioners for chemical releases and exposure analysis.^23^ Meyer et al.^24^ used a tree-based ML regressor for estimating chemical releases from manufacturing processes. Ring et al.^25^ developed predictive algorithms to obtain the probabilities that a chemical might be associated with a near-field exposure pathway (i.e., consumer exposure). Based on existing data, Franzosa et al.^26^ gained valuable information on key events or pathways that may result in adverse effects on human tissues. Isaacs et al.^27^ used ML classifiers for obtaining chemical functions and weight fractions in consumer products. Cha et al.^28^ used a random forest regressor to predict the oxidant exposure during ozonation reactions. Fan and Xu^29^ implemented deep learning models for predicting the health risks of occupational exposure to toxic chemicals in coal mine workplaces. ML approaches and techniques have been used for the modeling, design, and prediction of energy systems, including the use of support vector machines to estimate carbon dioxide emissions from combined cycle gas turbine plants.^30^ Huang et al.^31^ explored the use of deep learning algorithms for air pollutant emission estimation. Tu et al.^32^ presented a hybrid modeling approach, named “cluster-based validated emission recalculation,” for predicting the emissions of greenhouse gases from the Great Toronto Area.

An ML model can use different type of predictors or features that are related to an output or response variable in a particular application domain (e.g., exposure analysis).^33^ Quantitative structure–activity relationship (QSAR) modeling has been widely used in medicinal chemistry and computational toxicology. QSAR modeling offers an in silico tool for the development of predictive models that relate various activity and property endpoints (i.e., response variables) of chemicals and molecular structure information.^34^ Beyond the computational toxicology field, ML QSAR models have been implemented in a wide range of topics. Phillips et al.^35^ used ML QSAR based on random forest for screening and identifying chemicals that can provide a specific function within products so that hazardous chemicals can be substituted for safer candidates. Song et al.^36^ developed an ML QSAR approach via artificial neural networks for performing a rapid life cycle impact assessment for chemicals. Holmquist et al.^37^ developed an ML QSAR for obtaining characterization factors for simplified life cycle impact assessment. Holmquist et al.^37^ developed a framework that can be used to rapidly assess per- and polyfluoroalkyl substances.^38^

The extensive number of techniques and strategies for ML QSAR modeling makes it a viable alternative for extending the CFA described above to chemicals that are either not reported to regulatory primary data sources or are new to the market. Based on the work developed by Hernandez-Betancur et al.,^22^ this work utilizes the PRTR_transfers database to develop ML QSAR models for predicting potential EoL activities and identifying EoL exposure scenarios for chemicals found in industrial material transfers to off-site locations for further EoL management. Thus, allocating EoL chemicals flows by the predicted EoL activities and their operating conditions establish potential environmental releases and exposure pathways at the EoL stage.^39^ In addition to chemical descriptors from the PRTR database, the ML QSAR models, hereinafter called quantitative structure-transfer relationship (QSTR) models, use the generator industry sector, the EoL flow transfer amount, the environmental stringency by country and year, the chemical unit price, and the industry gross value added by country and year as predictors (i.e., features or input parameters) to predict the potential EoL activities for chemicals outside the PRTR_transfers database. The QSTR models can be incorporated into the Markov random field described by Hernandez-Betancur et al.^20^ to predict the model factors or “probabilities” that relate to the strength of the relationship between generator industry sectors and off-site EoL activities. Moreover, these work outcomes can be complemented by estimating environmental releases, EoL CFA fractions, and incorporating sustainability indicators for EoL exposure assessment.^40^

## Methodology

A series of steps that are a part of the life cycle of an ML model and system were necessary for the developing QSTR models to predict potential EoL activities and material allocation for industrial chemical transfers to off-site locations for further EoL management. The methodology consists of five main steps. The first step collects and enriches the data to describe crucial factors that will influence decision-makers to determine potential off-site EoL activities for the chemicals of concern. The second step focuses on processing the data before feeding them into the QSTR models. The third step builds the QSTR models. The fourth step uses multi-criteria decision-making to select the data preprocessing pipeline needed to obtain high QSTR model performance. The final step is the hyperparameter (e.g., number of trees in a random forest) optimization and tuning to obtain the best possible performance for the QSTR models.

### Data Sources

**Figure 1:**
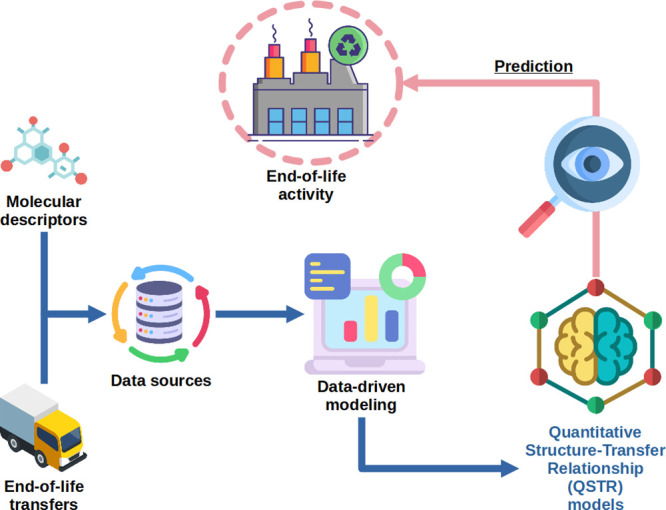
Use of structure-based classification models for predicting potential EoL activities for EoL chemical off-site transfers.

The data used to create the QSTR models include predictors that have an impact on the likelihood that an EoL activity will occur for a chemical, as illustrated in Figure 1 (e.g., environmental policy). The main data source to develop the QSTR models is the PRTR_transfers database built by Hernandez-Betancur et al.^22^ Data from siloed and publicly accessible database systems belonging to OECD member nations were standardized and harmonized and stored in this database.^41^ This database’s information is chemical-centric, focusing on individual chemicals rather than the total quantity of hazardous or non-hazardous wastes. The aforementioned is appropriate for evaluating chemical exposure and risk, as toxicologists usually evaluate chemicals separately instead of mixtures. This database provides LCI facility-level data that include off-site transfers from facilities and businesses manufacturing the chemical, processing it as a reactant or intermediate, incorporating it into formulations, mixtures, or reaction products, incorporating it into articles, and using it industrially. The PRTR_transfers database has the information to describe the generator industry sectors, EoL activities or off-site transfer scenarios, the quantity of chemicals in the off-site transferred flows (in kg/year), and the chemical substances. The database contains 72 industry sectors, 643 chemical substances, and a total of 3,116,211 records. The PRTR_transfers database contains 10 EoL activities or off-site transfer classes: surface impoundment, sewerage, destruction, energy recovery, landfill, other disposal, other treatment, recycling, storage, and underground injection.

Data associated with the chemical unit price and the gross value added by an industry sector are collected to represent the economic bottom line of sustainability since the economic sustainability dimension may affect the occurrence of an EoL activity for a chemical.^21^ These economic considerations are crucial because a company can be willing to recycle a high-value chemical but destroy a low-value one. Moreover, a business with high income or sales, which are related to the gross value added,^42^ may afford to operate an EoL activity to handle or abate hazardous chemicals and/or wastes.^43^ Using the CAS numbers for the chemicals, their unit prices (in USD/g) are retrieved automatically from e-commerce sources like SciFinder, Amazon, Alibaba, and Fisher Scientific. Due to the massive inventory of chemical suppliers around the world, these systems, particularly SciFinder, are utilized as search engines, making it easier to find chemical prices.

The gross value added by the industry sector is obtained from the OECD statistics,^44^ considering the year and country of the reporting record. If the gross value added by industry and country is not found for a particular year, it is imputed by using linear extrapolation and country data. In addition, around 400 molecular descriptors are obtained from RDKit cheminformatics software via its Python API.^45^ However, before obtaining the descriptors, each chemical’s SMILES is required. SMILES is a notation to encode information on the molecular structure to be understood by a computer.^46^ Through automation, the SMILESs are obtained from the APIs of the US National Library of Medicine and PubChem.

The environmental context that may affect the probability of an EoL activity occurrence is represented by the OECD environmental policy stringency index obtained from OECD statistics.^44^ This index is a country-specific and internationally comparable measure of the stringency of environmental policy.^47^ In cases where the index is not found for a country, linear extrapolation, and the country data are used for item imputation. Linear extrapolation has drawbacks, including the inability to take causal elements into account in the calculus-based observations and the failure to take into consideration qualitative values that can affect future values.^48^ Gross value added and environmental stringency indexes reported over time can help to expand the QSTR model application domain, particularly for countries that are not part of the PRTR_transfers database, but their industry sector economy and environmental regulation could be represented by any reporting country and/or sector in previous years.

### Data-Driven Modeling

Instead of being based on any environmental decision-making foundation, some components of the methodology are mathematical operations linked to transforming the data into a machine-readable structure to be understood by the models or performing hyperparameter optimization. The crucial steps for environmental science practitioners to make a more informed and appropriate decision will be highlighted to maintain the focus on the advantages in the context of sustainable chemistry and engineering. The manuscript’s Supporting Information (SI), which goes into greater detail regarding the methodology, is available to readers.

The information in the PRTR_transfers database is imbalanced, i.e., sample data has more entries linked with an EoL activity than others. For instance, there are more records for landfills than for storage operations in the PRTR_transfers. QSTRs are classification models that scan EoL operations for industrial chemical and support specialists in order to quickly identify potential and high-priority activities that need additional analysis. If the PRTR_transfers imbalance is not addressed, the QSTRs may classify EoL activities incorrectly for the parameters specified by model users.^49^ The above would be troublesome because for EoL activities with few samples, the QSTR might suggest that an EoL activity would not occur when it does (false negative). The contrary may happen for activities having majority of the samples, indicating that the activity would occur when it does not (false positive).^50^ Despite the fact that both scenarios are unpleasant, the false negative scenario would be the worst since no proper managerial techniques or equipment would be employed to control the exposure to dangerous chemicals that would damage human health and the environment.^51^ As a result, this contribution applies ML techniques to address the data imbalance; for more information on those techniques, see the SI.

In the context of QSAR modeling, the OECD suggests using, when it is possible, models that enable getting a mechanistic explanation of the model results. It means that it is better to use a model whose complexity enables us to determine the predictors/variable that are more related to a response or target variable (i.e., EoL activity in our case) in an environmental context.^52^ Thus, this research employs a random forest classifier (RFC), which has been demonstrated to be a high-performance algorithm in the field of QSAR,^53,54^ and it can supply information about the variable’s importance.^55^ In addition to the ML models, this contribution explores three modeling strategies: one-vs-all, multi-class classification, and multi-label classification. EoL activities cannot take place simultaneously according to the multi-class classification.^56^ Contrary to multi-class classification, multi-label classification allows for the consideration of one or more activities occurring simultaneously for a chemical, which is possible given that the PRTR_transfers contains annually reported LCI data.^57^ One-vs-all or binary classification creates a QSTR for each of the 10 EoL activities.

In the QSTR experiment tracking, this contribution aims to investigate not only models that can deliver precise and high-performance results at a model low-complexity level but also QSTRs that can provide predictions in a reasonable time and without demanding high costs of storage in case of future use by environmental policy-makers and businesses. Twenty percent of the 30% records from the PRTR transfers database were used in the external QSTR validation. Experiments are carried out to track QSTR performance using baseline values for the RFC, and dimensionality reduction techniques for the three modeling strategies are selected.^58^ The models are optimized to improve their performance as needed, considering factors such as a minimum *f*_1_-score value of 70%, the possibility of improvement across optimization iterations, and an optimization time budget (see the SI). For further information on the methodology, refer to the SI.

## Results and Discussion

This section presents the results obtained following the methodology. The ML predicting model developed in this work should be considered acknowledging its assumptions and limitations, especially since only 30% of the PRTR_transfers database records are used. A data-centric paradigm is needed for developing robust ML models to work in the real world, i.e., keeping the model structure and workflow, while the data are enhanced.^59^ A public GitHub repository contains the scripts and notebooks developed for the QSTR model development presented in this manuscript (see the SI). For more details on the outcomes and experiment tracking, see the SI.

### External QSTR Model Validation

The results for the QSTR models following hyperparameter optimization for the three modeling approaches are shown and discussed in this section. Table 1 summarizes the scores for the QSTR models obtained. If used to predict EoL activities, the created QSTR models based on the multi-class and multi-label classification will underperform. However, the one-vs-all strategy-built QSTR was expected to be a good alternative for aiding in mapping EoL activities and, subsequently, the operating conditions related to exposure at the EoL stage.

**Table 1:** Obtained Scores for the External Validation of the QSTR Models Developed under the Three Modeling Strategies

modeling strategy	target EoL activity	test accuracy	test *f*_1_
multi-class classification	all	0.29	0.24
multi-label classification	all	0.41	0.46
one-vs-all	surface impoundment	0.97	0.97
	destruction	0.74	0.76
	energy recovery	0.80	0.82
	landfill	0.67	0.69
	other disposal	0.75	0.75
	other treatment	0.72	0.73
	recycling	0.72	0.73
	storage	0.93	0.93
	underground injection	0.93	0.94
	sewerage	0.73	0.75

The difference in classification scores between multi-label and multi-class highlights an interesting conclusion that the LCI data from the PRTR transfer database would make it possible to construct QSTR models that predict potential outcomes for an industrial and hazardous chemical over an annual time horizon. Due to assumptions and uncertainty propagation, it would not be a good idea to attempt to adjust the time-scale for the PRTR transfer data.^60^ Environmental government agencies typically use annual data to evaluate chemical exposure and risk; therefore, applying and integrating the QSTR models into these types of evaluations will not present any hindrance.^61^ Except for the landfill QSTR, all of the QSTR models created using the one-vs-all technique have *f*_1_-scores that are higher than the targeted cutoff of 70%. The aforementioned indicates that if the QSTR models are used as is, landfill QSTR may provide issues with accurately predicting if landfill would occur for some chemicals. Nevertheless, the landfill QSTR’s hyperparameter tuning results indicate that this model might still keep becoming better even if a longer tuning time is given (see Figure S2a in the SI). Furthermore, it is important to remember that not all of the PRTR_transfers data were used for running ML experiments due to computer power limitations.

**Figure 2:**
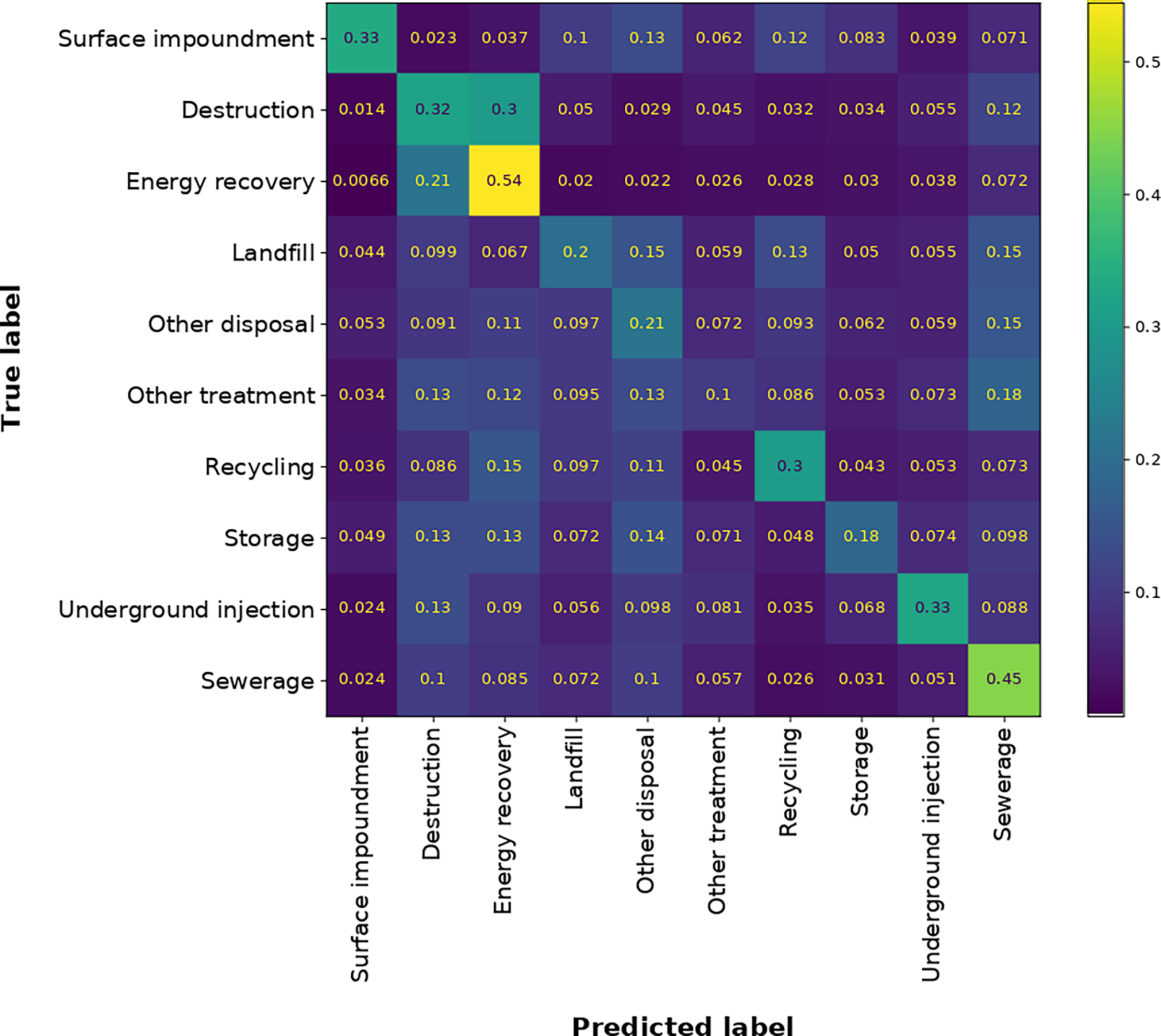
The confusion matrix for the QSTR model developed under the multi-class classification strategy. This picture was obtained for the model’s external evaluation on the test dataset. The numbers inside the squares represent the ratio between the predicted sample labels for EoL activity on the *x* axis and the total true sample labels for EoL activity on the *y* axis. A dark blue color is for lower ratios, while a yellow color is for higher ratios.

Figure 2 presents the confusion matrix for the multi-class classification. The *y* axis represents the true label for a test sample, and the *x* axis represents the label that is predicted by the multi-class classifier. The numbers inside the matrix are the relationship between the number of samples in the row predicted with the label on the *x* axis and the number of total samples with the label on the *y* axis. For example, 54% of the energy recovery samples (yellow square in Figure 2) were correctly predicted as belonging to energy recovery. The correctly predicted EoL activities range from 10 to 54%, as shown by the matrix diagonal. The percentage of 21% represents the highest misclassification that corresponds to samples whose labels are truly energy recovery but are misclassified as destruction. Destruction contains EoL activities related to incineration. The similarity between the nature of energy recovery and incineration can lead to potential misclassification. Something similar occurs with other treatments for which the QSTR model developed by the multi-class classification strategy misclassifies 18% of the samples as sewerage.

In summary, the multi-class classification strategy cannot provide a good performance because it assumes the EoL activities are mutually exclusive, i.e., they cannot occur at the same time for a chemical. The aforementioned is made worse when taking into account any phenomenological parallels that EoL activity operations may have because the multi-class QSTR could not accurately identify or differentiate between EoL activities. For instance, in the USA, “to claim that a combustion operation is used for the purpose of energy recovery and not for treatment for destruction, the chemical must have a significant heating value and must be combusted in an energy recovery unit such as an industrial boiler, furnace, or kiln”.^62^ Thus, the “incineration/insignificant fuel value” category represents that the chemical goes into a “legitimate energy recovery unit”, but it does not “contribute to the heating value of the waste”.^62^

As a conclusion to this section, although while RFC natively handles multi-label classification problems, it is preferable to combine RFC with any specialized method to solve multi-label problems, such as label powersets or classifier chains.^63^ The models developed here are not conclusive but can be a starting point for future development, indicating the one-vs-all strategy should be implemented for the development of QSTR models since it outperforms the other two strategies. Moreover, a multi-label classification strategy complemented with a transformation problem technique like label powersets can help with the QSTR model by working only with one model instead of 10 as in the case of the one-vs-all strategy.

### QSTR Model Implication and Connection with Other Frameworks

The QSTR shows that is it possible to extend the framework developed by Hernandez-Betancur et al.^18^ and Hernandez-Betancur et al.^22^ The QSTR can predict the potential EoL activity that is part of the EoL exposure scenario to a chemical that is transferred to an off-site location for further management. The above can especially be done for chemicals that are not in the primary data sources but are part of the QSTR applicability domain (see the SI for model deployment). In addition, after identifying the potential off-site EoL activity for a chemical, it would be possible to connect this result with the proposal described by Hernandez-Betancur et al.^19^ and perform the CFA, including aspects of the pollution abatement technologies. Finally, the QSTR models can provide the factors for establishing a relationship between the generator industry sector and the EoL activity in the Markov random field developed by Hernandez-Betancur et al.^20^ to outline the relationship between the entities through the EoL management chain and recycling loop.

**Figure 3:**
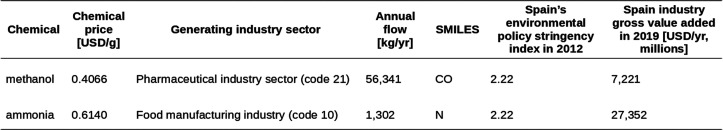
Input parameters for the application of the QSTR models to predict potential EoL activities for chemicals.

For more in-depth detail, Figure 3 contains the input parameters to the 10 QSTR models developed under the one-vs-all strategy (see the SI). The chemicals (i.e., methanol and ammonia), the sectors, and the annual chemical flows in Figure 3 are taken from two case studies developed by Hernandez-Betancur et al.^19^ The chemical price corresponds to the mean values found in SciFinder, and the SMILES are retrieved from PubChem. Moreover, the environmental policy stringency index and industry gross value added for Spain are obtained from the OECD statistics. Those values correspond to hypothetical cases of potential values that could be fed into the QSTR models to obtain predictions. Using the above values, the probability that an EoL activity will occur is predicted, as shown in the second and third columns in Table 2. For example, under the input parameters in Figure 3, methanol has the highest chance of being transferred to an off-site location for destruction, while ammonia goes to sewerage (see Table 2). The aforementioned findings suggest that environmental practitioners should concentrate on in-depth investigation of circumstances linked to the EoL activities for the chemicals, i.e., destruction for methanol and sewerage for ammonia, respectively.

**Table 2:** Prediction of EoL Activities and Probabilities Based on the 10 QSTR Models and the Mean Annual Mass Fractions Obtained Via Monte Carlo

	probability		mean annual mass fraction	
EoL activity	methanol	ammonia	methanol	ammonia
surface impoundment	0.28	0.42	0.0686	0.1044
destruction	0.52	0.39	0.1270	0.0969
energy recovery	0.45	0.35	0.1100	0.0871
landfill	0.38	0.48	0.0930	0.1196
other disposal	0.37	0.29	0.0905	0.0721
other treatment	0.36	0.37	0.0880	0.0920
recycling	0.32	0.28	0.0783	0.0697
storage	0.41	0.49	0.1002	0.1218
underground injection	0.49	0.32	0.1198	0.0796
sewerage	0.51	0.63	0.1246	0.1567

The probabilities in Table 2 could be used as the model factor or “probability” values describing the relationship strength between the EoL activity and generator industry sector nodes in the Markov random field proposed by Hernandez-Betancur et al.^20^ Using the Monte Carlo method, the local relationship between the two nodes can be described, providing the annual mean mass flows and, subsequently, the annual mean mass fractions, as shown in the fourth and fifth columns in Table 2. Hence, these fractions give the annual flows that can be connected to the framework described by Hernandez-Betancur et al.^19^ to perform CFA and allocate downstream of the pollution abatement unit technologies.

Considering chemical exposure assessment and environmental and human health impact reduction as upfront criteria to develop more sustainable processes, the proposed QSTR models could assist stakeholders in predicting potential EoL pathways of material flows generated during the chemical manufacturing life cycle stage. Therefore, at the early stages of process development, decision-makers can check if a new synthesis of chemical process may have undesirable EoL pathways and impacts based on chemical structure consideration, business location, and industrial activity. Thus, they will be able to select appropriate pollution abatement technologies, establish risk management procedures, and use source reduction techniques.^64^ Additionally, stakeholders can use the QSTR models to pinpoint potential EoL activities to consider the monetary and environmental costs of pollution control systems related to the management of chemical production releases. Instead, sustainable chemistry and engineering solutions can be evaluated to improve the chemical production sustainability and its impact when extending the analysis outside the chemical production facility gates.^65^

## Conclusions

This work contributes to the development of QSTR models, which are ML models using a QSAR modeling approach. The QSTR models can predict the probability of an EoL activity for a chemical of industrial relevance that is transferred to an off-site location, thereby helping identify the EoL exposure scenario for a chemical. By leveraging data, the work can incorporate features into the QSTR models associated with the generator industry sector, the amount of the chemical involved in an off-site transfer, molecular descriptors, the gross value added by an industry sector, environmental policy stringency, and the chemical unit price. These features help the QSTR models gain insights into the context related to the decision-making of an EoL activity alternative. Environmental decision-makers can use the predictions from the QSTR model to give particular attention to those high-priority scenarios for chemical exposure and risk reduction.

This work shows that RFC can be used to develop the QSTR models and make high-performance predictions of the potential EoL activities. Future work should explore the application of the multi-label classification strategy with the implementation of any special technique for multi-label problems such as label powersets. A considerable risk of misclassification exists between EoL activities like destruction via incineration and energy recovery; hence, aspects related to their similarity should be thoroughly examined in future development. To better understand the decision-making context and distinguish between these different types of EoL activities, more features could be incorporated into QSTR models. These models can be used to estimate the mass fraction of a chemical that might be moved to an off-site location for EoL management. To accurately characterize the behavior of the EoL management chain and recycling loop, further work should go beyond just incorporating the QSTR models suggested here into a statistical graphical model like the Markov random field. As a result, it is possible to address and improve CFA and EoL exposure assessment, offering valuable implications for environmental regulatory decision-making as well as business decision-making in the potential life cycle of chemicals circulating in workplaces, homes, neighborhoods, and, generally, the global marketplace.

## Supporting Information Available

Additional information about the data-driven methodology and experiment tracking and findings (PDF)

## Acronyms

CFA: chemical flow analysis
EoL: end-of-life
FAHP: fuzzy analytic hierarchy process
FAMD: factor analysis of mixed data
LCI: life cycle inventory
ML: machine learning
MLSMOTE: multi-label synthetic minority oversampling technique
OECD: Organization for Economic Cooperation and Development
QSAR: quantitative structure–activity relationship
QSTR: quantitative structure-transfer relationship
RFC: random forest classifier
SMOTE: synthetic minority oversampling technique
