# Supplementary material for: Predicting Chemical End-of-Life Scenarios Using Structure-Based Classification Models
Source: ACS Sustain Chem Eng. 2023 Feb 24;11(9):3594–602. doi: 10.1021/acssuschemeng.2c05662 (PMC9993395; doi:10.1021/acssuschemeng.2c05662)
Supplement: Supplementary file 1 — sc2c05662_si_001.pdf [file sc2c05662_si_001.pdf]

Supporting information

Predicting chemical end-of-life scenarios using structure-based classification models

Jose Hernandez-Betancur[1], Gerardo Ruiz-Mercado[2,3], Mariano Martin[1*]

[1] Department of Chemical Engineering, University of Salamanca, Salamanca, 37008, Spain

[2] Office of Research & Development, U.S. Environmental Protection Agency, Cincinnati, OH, 45268, USA

[3] Chemical Engineering Graduate Program, Universidad del Atlántico, Puerto Colombia, 080007, Colombia

[*] Corresponding author:

E-mail: mariano.m3@usal.es

Address: Plz. De los Caídos 1-5, Salamanca, 37008, Spain

Number of pages: 18

Number of tables: 3

Number of figures: 5

S1. GitHub repository for data-driven modeling

The following link contains the information for accessing the data-driven modeling scripts and notebooks:

https://github.com/jodhernandezbe/PRTR_transfers/tree/data-driven

https://github.com/USEPA/PRTR-QSTR-models/tree/data-driven

Data preparation pipelines	https://cutt.ly/8D6iex6<br>https://cutt.ly/6Cwlscj
Multi-class classification notebook	https://cutt.ly/hD6uNCd<br>https://cutt.ly/1CwlkzW
Multi-label classification notebook	https://cutt.ly/HD6u1Wi<br>https://cutt.ly/PCwlzJa
One-vs-all notebook	https://cutt.ly/8D6u9ug<br>https://cutt.ly/xCwlvp5
Base result for multi-class classification	https://cutt.ly/SD6ioZL<br>https://cutt.ly/ECwlQHx
Base result for multi-label classification	https://cutt.ly/wD6ikTr<br>https://cutt.ly/OCwlRxK

Base result for one-vs-all (over-sampling)	https://cutt.ly/8D6icns<br>https://cutt.ly/kCwlYZV
Base result for one-vs-all (under-sampling)	https://cutt.ly/RD6iRpc<br>https://cutt.ly/vCwlOIM
Figures for multi-class classification	https://cutt.ly/UD6iAHD<br>https://cutt.ly/ZCwlAHj
Figures for multi-label classification	https://cutt.ly/qD6iHwa<br>https://cutt.ly/xCwlFIF
Figures for one-vs-all	https://cutt.ly/3D6iLFu<br>https://cutt.ly/DCwlHOW

S2. Methodology

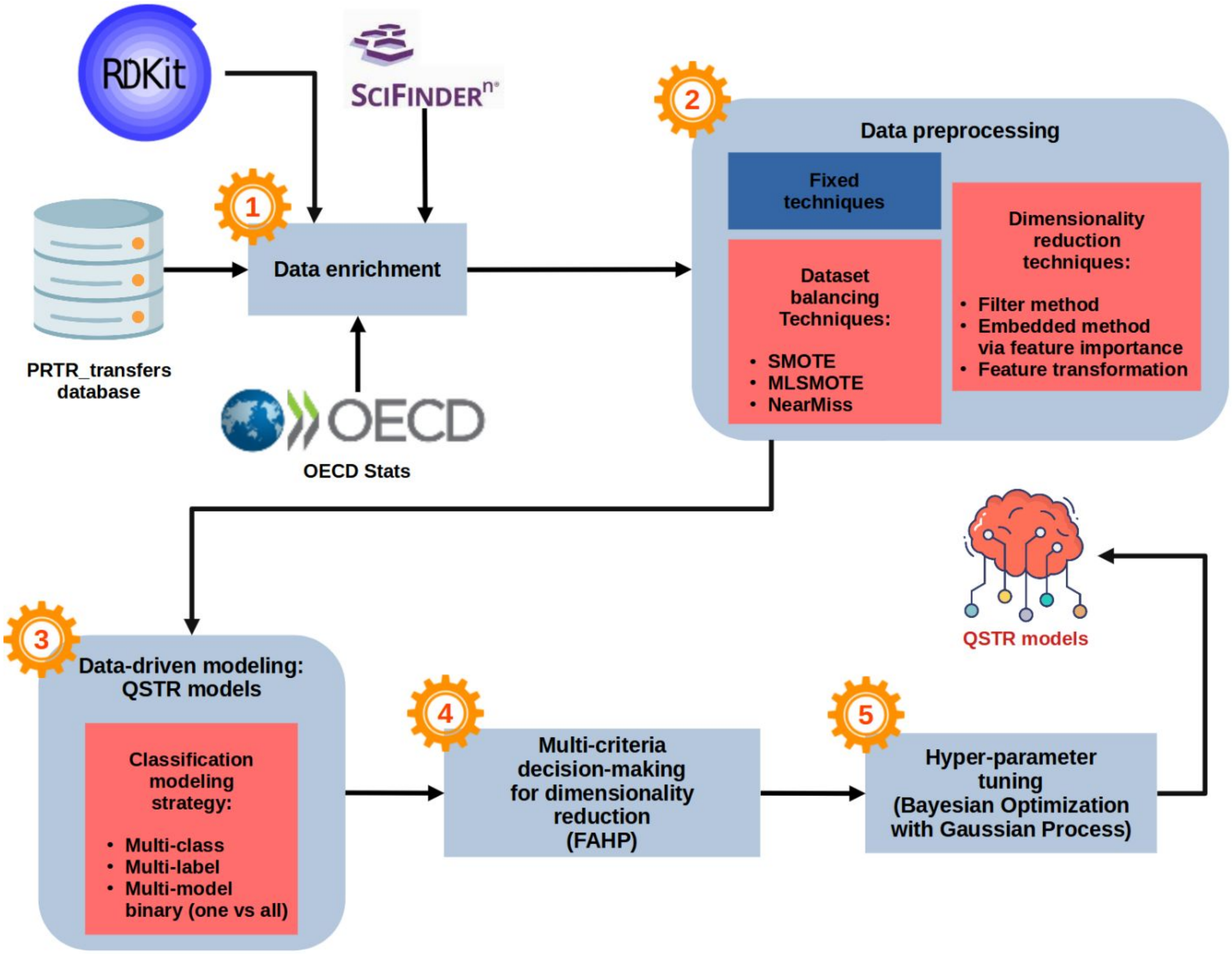

Figure S1. An overview of the workflow for developing QSTR models to identify potential EoL activities or for off-site transfer scenarios for chemicals.

S2.1. Data preparation

Like with any ML model development, developing QSTR models requires preparing the data before being fed into the models. In addition to dropping duplicate records, the following considerations are taken as part of the data preparation:

1. Imputation of missing values is based on a "lazy" method, where the statistical mode is used for integer features and the median for float ones.
2. Features are normalized by using min-max scaling that does not depend on whether the feature is normally distributed[1]. The scaling feature can help to accelerate model training convergence and improve model performance[2].
3. The presence of outliers is beneficial for knowledge discovery, but it can cause learning problems for both ML and QSAR models[3]. Distance-based outlier detection techniques can perform well for scattered real-world data[4]. However, distance-based outlier detectors may be computationally expensive in large datasets. Due to the above, this work implements a tree-based method called Isolation Forest that may be faster than distance-based methods and perform properly under its default parameters[5].
4. The PRTR_transfers database has information about chemical groups[6]. This work keeps the chemical groups and imputes the molecular descriptor values by using the mean value excluding outliers via z-score.
5. The discretization of continuous features (e.g., transferred material flows) can help to improve the performance of ML models[7]. Here, the transfer flows are discretized, i.e., mapped to finite values or intervals. The number of intervals for discretization is 10 balanced intervals split by quantiles, ensuring an equal number of samples for each interval.

6. For EoL activities or off-site transfer classes, the PRTR_transfers database is unbalanced[6]. That means there are not the same number of samples associated with each EoL activity. To avoid miss-classification due to dataset imbalance[8], ML techniques are applied to balance the training dataset. The balance technique depends on the modeling strategy used at the modeling stage. This contribution uses three strategies that are multi-class classification, multi-label classification, and one-vs-all. For multi-class classification, the Synthetic Minority Over-sampling Technique (SMOTE) is implemented[9]. For multi-label classification, an extension of SMOTE called Multi-Label SMOTE (MLSMOTE) is applied[10]. For the one-vs-all strategy, the trails are run first using an under-sampling technique via NearMiss[11] and second, implementing SMOTE, and their results are compared.

7. High-dimensional data can cause overfitting problems during the ML learning process[12, 13] leading to a poor generalization of the model predictivity for new real-world data. In addition, from ML operations, reducing the ML model dimensionality can reduce the storage infrastructure needed to deploy the model and avoid feature redundancy[14]. Many dimensionality reduction techniques can be found in the literature, classified into different categories according to their taxonomy and functionality[15]. For the three modeling strategies, three different dimensionality reduction techniques are considered. Two of the dimensionality reduction techniques are based on feature selection. These techniques are only applied to the molecular descriptors before removing those whose variance is 0 (i.e., constant across the dataset) and variance is 0-0.01 (i.e., quasi-constant values across the dataset). The first feature selection technique used is univariate feature selection, and the second one is a wrapper method using a random forest classifier (RFC)[16]. For both feature selection techniques, only the best 20% of

molecular descriptors are kept (i.e., those explaining the variation of Y). The third technique is based on feature transformation and is called Factor Analysis of Mixed Data (FAMD). FAMD is like the well-known principal component analysis, but unlike principal component analysis, it can be implemented over datasets containing both numerical and categorical features without requiring feature encoding[17]. If FAMD is used, it will be implemented over all the dataset features and not only the molecular descriptors. In addition, the total transformed features kept correspondence to the ones that explained 95% of the variance.

8. If a feature selection is applied for dimensionality reduction, the categorical features like the industry sector are coded using one-hot encoding. One-hot encoding is a widely used technique for mapping categorical features to numeric space without inducing any ordinal importance in the transformed features[18].

S2.2. Data-driven modeling and modeling strategies

Any available ML model in the literature (e.g., gradient boosting classifier) could be tested to build up the QSTR models. Due to a large number of existing ML models, data-driven modeling could be a time-consuming and iterative task. Therefore, model selection is part of the ML operations[14]. At this point, the state of the art in developing QSAR models is relevant for accelerating the modeling process and reducing iterations. The random forest has been demonstrated to be a high-performance algorithm in the field of QSARs[19, 20]. In addition, this algorithm can handle a very large number of input variables without overfitting, and it can provide information about how important a variable is, thereby helping to explain the result if required[21]. As this work is looking to provide an estimate of how probable an EoL activity is for a chemical of concern, the performance of an RFC would be tested to obtain the QSTR

models. As common in ML model development, the first RFCs in step 3 in Figure S1 is done using a base RFC, i.e., a QSTR model.

In addition, three modeling strategies are implemented and tested. The first strategy is the multi-class classification. Multi-class classification considers that the EoL activities are mutually exclusive, i.e., they cannot occur at the same time for a chemical [22]. The second strategy is multi-label classification. Unlike multi-class classification, multi-label modeling considers that the EoL activities are non-mutually exclusive. That means that more than one EoL activity can occur for a chemical at the same time[23]. Not all the ML models support multi-label classification natively, but RFC is part of the group of ML models that do[24]. However, in some cases, a single generalized model is not enough for dealing with multi-label problems, and an alternative strategy is to split the multi-label problem into multiple independent models according to the number of existing target classes (10 EoL activities or off-site transfer classes)[25]. The final strategy is multi-model binary classification or one-vs-all, where a QSTR model is developed for each EoL activity (i.e., 10 QSTR models are developed).

Due to the computer power limitation, only 30% of the records in the PRTR_transfers database are used for the trials, which is also a reasonable percentage considering the experimental and exploratory nature of this work and that many modeling pathways have to be tested. To capture the statistical distribution of the EoL activities or off-site transfer classes (i.e., QSTRs response variable), this dataset is drawn by stratified splitting so that the dataset keeps the same proportion of samples for each EoL activity as the one in the PRTR_transfers database. Like with any ML model, the development of the QSTRs requires an internal and external validation of the model's performance[26]. The

dataset is further split stratified into training (internal validation set) and test (external validation set) datasets, taking 20% of the 30% records being used for external validation of QSTRs.

The internal validation uses 5-fold cross-validation that not only helps in model selection tasks but also identifies a potential underfitting and overfitting behavior[27]. In addition, QSAR model development usually implements a Y-randomization technique in the internal validation to ensure that the successful performance of the classifier is not due to a chance correlation between the model features and function[26]. However, Y-randomization has implementation variants[28] and this work utilizes the one described by Ponzoni et al. [29], using only 10 permutations in each trial[30]. To measure the performance of QSTR models developed under multi-class classification and one-vs-all strategies, the accuracy and error metrics are used[31], in conjunction with the weighted f1-score[32]. For a multi-label classification strategy, the prediction for a record is a set of labels so that the prediction could be fully correct, partially correct, or fully incorrect. Therefore, for this strategy, the accuracy, f1-score, and 0/1 loss metrics are utilized and implemented in the way described by Sorower[33]. In this manuscript, a QSTR model is considered to perform well when it satisfies two criteria. The first criterion is that the accuracy is greater or equal to the desired threshold of 70% since models with an accuracy of at least this value are considered good performers. The second criterion considers that the mean cross-validation error or 0/1 loss must be less than the lower bound of Y-randomization error or 01/loss ($\overline{E}_{CV} < \overline{E}_Y - \sigma_Y$).

S2.3. Multi-criteria decision-making: dimensionality reduction technique

As presented above, three different dimensionality reduction techniques are applied to each modeling strategy (e.g., multi-class classification). As shown in Figure S1, multi-criteria decision-making is utilized to select the

technique that leads to the best performance for a modeling strategy. Fuzzy Analytic Hierarchy Process (FAHP) is used to address this multi-criteria problem, considering triangular fuzzy numbers[34, 35]. A total of five criteria are considered by the FAHP. The first criterion considers whether the 5-fold cross-validation results show over-fitting, under-fitting, or optimal-fitting. The first FAHP criterion is supported by a non-parametric hypothesis test called the Wilcoxon Signed-Rank Test with a 5% level of significance, for determining a ranking of four levels: optimal-fitting, over-fitting (high variance), under-fitting (high bias), and under-fitting (high bias and high variance). Wilcoxon Signed-Rank Test is equivalent to the dependent t-test and it is used to compare two sets of scores that come from a set of matched samples, as the case for 5-fold cross-validation scores[36]. The second criterion considers whether a high-performance or good fitting is obtained based on the accuracy. The third criterion takes the time per sample size consumed for obtaining the 5-fold cross-validation and Y-randomization results. This criterion is associated with the time a deployed or serving QSTR model would take to provide a prediction. The fourth criterion considers whether $\overline{E}_{CV} < \overline{E}_{Y} - \sigma_{Y}$. The final criterion is whether the dimensionality reduction technique enables interpreting the model results (e.g., the key variables in selecting an EoL activity). For example, FAMD reduces dimensionality by transforming the original input variables (e.g., chemical unit price) into new orthogonal variables.

S2.4. Hyper-parameter tuning

As shown in Figure S1, after FAHP, the QSTR models developed with the dimensionality reduction technique chosen by FAHP move to hyperparameter tuning (choosing a set of optimal parameters whose values control the learning algorithm process) or optimization to enhance the performance of the QSTR models for each strategy. Grid search is an optimization technique widely used by ML practitioners. This method runs through a whole grid of

values for the hyperparameters and, in the end, selects the best performing combination[37]. Another method used for optimization is random search, which, unlike grid search, randomly draws hyperparameter values and trials on the grid [38]. Bayesian optimization has also been utilized for searching for a high-performance hyperparameter combination. Bayesian optimization is a sequential model-based optimization algorithm that uses the previous iteration to decide the best hyperparameter value candidates[39]. Thus, unlike grid and random searches, Bayesian optimization trials are not independent of each other, and it selects hyperparameters in an informed way. Bayesian optimization requires a surrogate model to represent an objective function that can be sampled efficiently[39]. A common technique to obtain a surrogate model is the Gaussian Process, which constructs a joint probability distribution over the hyperparameters, assuming a multivariate gaussian distribution[40].

This contribution uses a Bayesian optimization with Gaussian Process to tune hyperparameters because it can speed up the hyperparameter tuning process due to its informed way (based on previous trial results) to select a trial. The RFC hyperparameters to optimize are (i) the minimum number of samples required to be at a leaf node, (ii) the minimum number of samples required to split an internal node, (iii) the maximum number of features the RFC is allowed to try in an individual tree, (iv) the number of trees in the RFC, (v) the longest path between the root node and the leaf node, (vi) the criterion to measure the quality of a split (e.g., information entropy), and (vii) whether bootstrapping is implemented (i.e., random sampling with replacement). These hyperparameters were chosen for tuning due to their impact on RFC performance[41]. The optimization looks to minimize the negative of the mean 5-fold cross-validation f1-score. The optimization is stopped if the model reaches a plateau after 5 trials with a delta of less than 1e-4. In addition, a time budget (after the initial random trials) of 2 hours is considered for multi-class

classification and one-vs-all strategies, while 4 hours is used for multi-label problems. These time budgets are selected considering the strategy complexity and computational power demanded and available for the optimization tasks. The optimization is also stopped when the negative of the mean 5-fold cross-validation f1-score reaches a value less or equal to -70%.

S3. Additional case study results

S3.1. Base SQTR model development and FAHP

As normal in any ML model development, the first model testing is done on a base model. Internal validation results for the base multi-class classification QSTR model indicate univariant feature selection is the best option for dimensionality reduction. This technique offers a trade-off between the five criteria considered by the FAHP. However, the multi-class QSTR model using this technique may not satisfy an accuracy value of at least 70% on the 3test dataset and it may not provide good predictions for new data due to over-fitting problems (see Section S3.1.1). Regarding the multi-label classification strategy, the wrapper method using RFC provides the best dimensionality reduction under the FAHP criteria. Unlike in multi-class classification strategy modeling, multi-label classification has a lower risk of over-fitting. However, like multi-class, the multi-label classification may not achieve an accuracy equal to or greater than 70% on the test dataset (see Section S3.1.2).

Table S1. 5-fold cross-validation and Y-randomization results for the base QSTR models developed using the one-vs-all strategy. The results are only for the cases selected by the FAHP for each of the 10 EoL activities. These results correspond to the data preparation workflows where SMOTE is applied.

Target EoL activity	Mean validation accuracy	Mean train accuracy	Mean validation f1	Mean train f1	Mean validation error ($\bar{E}_{CV}$)	Std validation error	Mean Y-randomization error ($\bar{E}_Y$)	Std Y-randomization error (σ_Y)
Surface impoundment	0.99	1.00	0.99	1.00	0.01	0.000599	0.50	0.002136

Destruction	0.81	0.95	0.81	0.95	0.19	0.001110	0.50	0.001784
Energy recovery	0.85	0.96	0.85	0.96	0.15	0.000668	0.50	0.001821
Landfill	0.70	0.92	0.70	0.92	0.30	0.001348	0.50	0.001158
Other disposal	0.82	0.95	0.82	0.95	0.18	0.001396	0.50	0.001761
Other treatment	0.77	0.94	0.77	0.94	0.23	0.000857	0.50	0.002237
Recycling	0.74	0.93	0.74	0.93	0.26	0.001260	0.50	0.001669
Storage	0.96	0.99	0.96	0.99	0.04	0.000375	0.50	0.001205
Underground injection	0.97	0.99	0.97	0.99	0.03	0.000434	0.50	0.002448
Sewerage	0.77	0.94	0.77	0.94	0.23	0.002125	0.50	0.001990

As described in Section S2.1., two different techniques for dealing with imbalanced datasets are used in the one-vs-all strategy, specifically under-sampling by NearMiss and over-sampling by SMOTE. When under-sampling is used for the development of the QSTR models based on the one-vs-all strategy, the Y-randomization shows that 21 out of 30 trials have the mean cross-validation error or 0/1 loss must be less than the lower bound of Y-randomization error or 01/loss ($\overline{E}_{CV} < \overline{E}_Y - \sigma_Y$). Thus, the classification may be due to a chance correlation between one or more descriptors and the target EoL activity (see Section S1). According to Y-randomization, SMOTE performs better than under-sampling via NearMiss because $\overline{E}_{CV} < \overline{E}_Y - \sigma_Y$ is achieved. Table S1 contains the results for the one-vs-all strategy using SMOTE. After the FAHP application, univariant feature selection is chosen as the best dimensionality reduction strategy for all the 10 QSTR models that are obtained via the one-vs-all strategy (one per target or EoL activity). All the selected QSTR models satisfy the criterion that $\overline{E}_{CV} < \overline{E}_Y - \sigma_Y$. However, the selected data workflows for landfill and recycling activities should be tuned to find a better hyperparameter set for improving their performance above the desired threshold (i.e., 70%). In addition, QSTR models associated with destruction, energy recovery, other disposal, other treatment, and sewerage activities should also be tuned to try to find hyperparameters to reduce the

difference between training and validation scores. Thus, a total of seven QSTR models built using the one-vs-all strategy should be tuned. In contrast, the remaining three models do not require further hyperparameter optimization to achieve the desired acceptance criteria.

S3.1.1. Multi-class classification

Table S2 presents the results obtained for the QSTR models that are developed by using the multi-class classification strategy. The table shows the internal validation results for the base QSTR model under this strategy. As shown in Table S2, the criterion that $\bar{E}_{CV} < \bar{E}_{Y} - \sigma_{Y}$ is satisfied for the models developed using the three dimensionality reduction techniques. Nonetheless, the mean f1 and accuracy score differences between the validation and training datasets shows that the QSTR model developed under this strategy may suffer from over-fitting. Thus, if these QSTR models are deployed, they may offer poor predictions and generalization for new data. In addition, the base QSTR model developed under this strategy may not satisfy that the accuracy value is at least 70% for the test dataset (i.e., under external validation). Moreover, the bright red color in Table S2 indicates that the dimensionality reduction selected under the FAHP five criteria is univariant feature selection. The reason for this selection is a trade-off between the scores and the time that may be required by the model to make predictions on new data.

Table S2. 5-fold cross-validation and Y-randomization results for the base QSTR model developed using multi-class classification strategy. The bright red color represents the results for the dimensionality reduction technique selected under FAHP.

Dimensionality reduction technique	Mean validation accuracy	Mean train accuracy	Mean validation f1	Mean train f1	Mean validation error ($\bar{E}_{CV}$)	Std validation error	Mean Y-randomization error ($\bar{E}_{Y}$)	Std Y-randomization error (σ_{Y})
Wrapper	0.45	0.80	0.44	0.80	0.55	0.000501	0.90	0.007999
Univariant	0.45	0.80	0.44	0.80	0.55	0.000975	0.90	0.000512
FAMD	0.4	0.77	0.40	0.77	0.60	0.002881	0.90	0.003332

Dimensionality reduction technique	Time [sec]	# Samples	# Bytes
Wrapper	614.02	486,440	322,996,160
Univariant	591.10	486,440	307,430,080
FAMD	204.08	59,070	29,298,720

S3.1.2. Multi-label classification

According to the results presented in Table S3, for multi-label classification modeling, the wrapper method using RFC is selected by FAHP. This decision may be mostly due to a trade-off between dimensionality reduction technique complexity, time that the model may require to make predictions on new data, and performance given f1 score and accuracy metrics. Like for multi-class classification, the criterion that $\overline{E}_{CV} < \overline{E}_{Y} - \sigma_{Y}$ is also satisfied. Moreover, the Y-randomization test shows that it is more difficult to obtain a successful QSTR model because of a chance correlation for multi-label classification than for multi-class one. The reason is that the 0/1 loss metric considers whether a sample is fully correct or fully incorrect. An important aspect to highlight is that the QSTR models developed using this strategy may have a lower risk of over-fitting than the multi-class classification strategy, as shown by the accuracy and f1 scores. In addition, as the mean validation accuracies are below 70%, it is possible that the accuracy score on the test dataset is even lower. Nonetheless, this final decision should be made after the hyperparameter optimization.

Table S3. . 5-fold cross-validation and Y-randomization results for the base QSTR model developed using multi-label classification strategy. The bright red color represents the results for the dimensionality reduction technique selected under FAHP.

Dimensionality reduction technique	Mean validation accuracy	Mean train accuracy	Mean validation f1	Mean train f1	Mean validation 0/1 loss ($\overline{E}_{CV}$)	Std validation 0/1 loss	Mean Y-randomization 0/1 loss ($\overline{E}_{Y}$)	Std Y-randomization 0/1 loss (σ_{Y})
Wrapper	0.67	0.98	0.71	0.98	0.46	0.001924	1.00	0.000323
Univariant	0.67	0.98	0.71	0.98	0.46	0.001416	1.00	0.000245

FAMD	0.68	0.98	0.72	0.98	0.45	0.001664	1.00	0.000266

Dimensionality reduction technique	Time [sec]	# Samples	# Bytes
Wrapper	3,902.78	286,112	222,022,912
Univariant	3,926.82	285,578	198,762,288
FAMD	5,938.61	285,888	198,978,048

S3.2. SQTR model hyperparameter optimization

After selecting the best dimensionality reduction strategies for each QSTR model, the QSTR performance must be improved by hyperparameter tuning. As described in Section S2.4, Bayesian optimization with a Gaussian process is used for tuning. Regarding the hyperparameter tuning for multi-class classification QSTR, its optimization achieves a parameter set, whose mean 5-fold cross-validation f1 is 0.47, which corresponds to an improvement of only 4% with respect to the base model obtained by univariate feature selection as a dimensionality reduction technique. The above means the QSTR accuracy on the test dataset is not expected to be equal to or greater than 70% (see Section S3.2.1.). For the multi-label case, the value of the optimization function does not reach a value different from the one obtained by the base model, and the accuracy on the test dataset is not expected to exceed 0.67, which corresponds to the mean validation accuracy (see Section S3.2.2.).

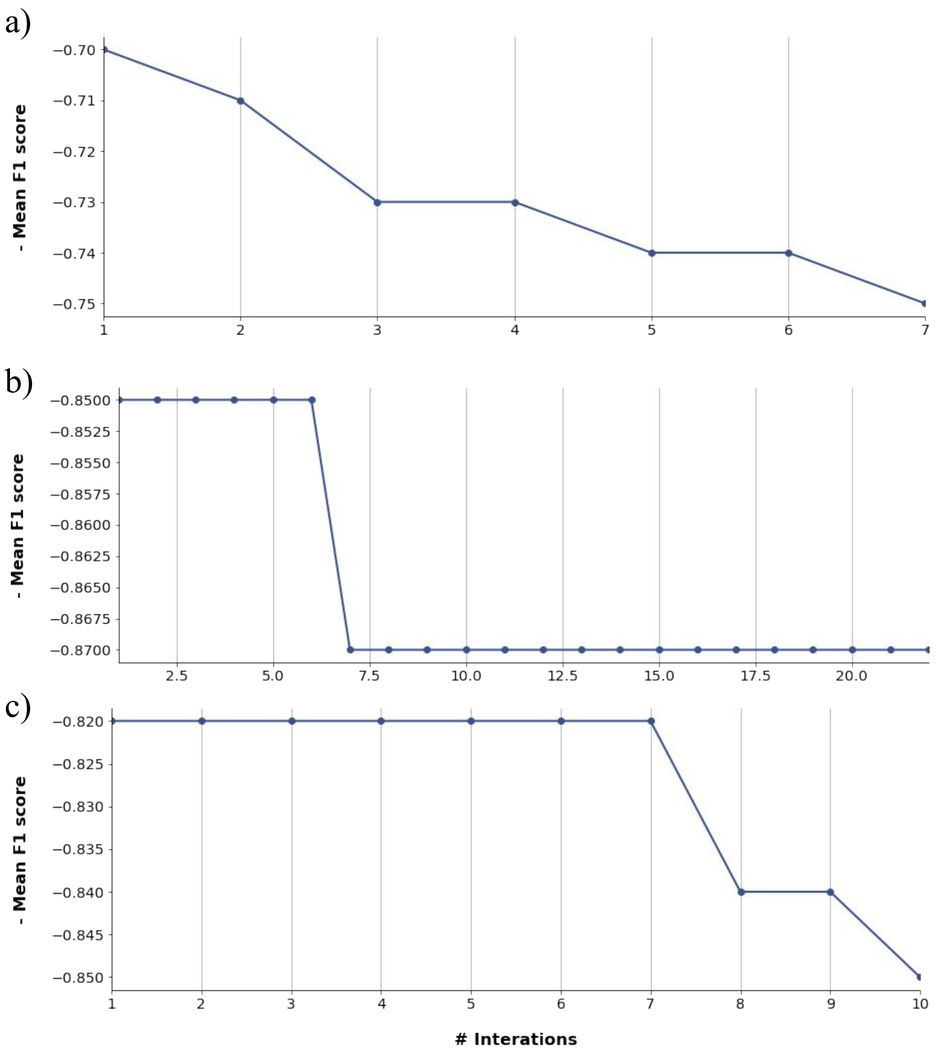

Figure S2. Convergence curves for the Bayesian optimization with Gaussian process for the QSTR. (a) It is for landfill developed under the one-vs-all strategy. (b) It is for energy recovery developed under the one-vs-all strategy. (c) It is for other disposal developed under the one-vs-all strategy.

Under the one-vs-all strategy, a total of seven QSTR models must be tuned, specifically the ones for landfill, recycling, destruction, energy recovery, other disposal, other treatment, and sewerage activities. Three out of eight convergence curves are selected because of their behavior and shape (see the link for the other ones in Section S1). The convergence curve shows only the best value for the global function in an iteration. Regarding the time required for tuning, the actual tuning time spent ranges from 136 seconds (for the sewerage QSTR model) to 1,278 seconds

(for the energy recovery QSTR model). The above means that for QSTR models like the one for sewerage, it is easier to achieve a desired objective function value in a short time, while for models like the one for energy recovery, it needs much more time to obtain a good-performance hyperparameter combination. The explanation is that, in some cases, it is difficult for the QSTR model to adequately discriminate the decision boundary between one EoL activity and another. For example, the QSTR model for energy recovery can have difficulties differentiating between some destruction activities associated with incineration because a combustion process is applied for both energy recovery and incineration activities. In addition, in some cases, some requirements should be satisfied from the point of view of environmental regulation so that a combustion activity can be considered energy recovery[89].

Figure S2a depicts the convergence curve for the landfill QSTR model. For this model, the convergence curve shows a downward trend. The last iteration indicates that for this model, it may be possible to obtain further improvement in the objective function if the time budget is increased. The – mean f1 score moves from -0.70 to -0.75, representing an improvement of around 7% of the mean f1 score with respect to one for the base landfill QSTR model in Table S1. The convergence curve shape for all the QSTR models developed under the one-vs-all strategy is not downward-trending. For example, the curves for the energy recovery and other disposal QSTR models show difficulty in finding a hyperparameter combination to improve the QSTR performance, as shown in Figure S2b and Figure S2c, respectively. The curve in Figure S2b shows that it is complicated for the energy recovery to achieve a better value for the objective function, even when the optimization takes around 1,278 seconds. As mentioned before, this problem can be because the QSTR model cannot find a decision boundary to separate energy recovery activities from the destruction ones via incineration, leading to an improvement in the performance of only 2% with respect to the base

model. As shown in Figure S2c, the QSTR models for other disposal activities can present similar behavior. The reason can be that a hazardous chemical has a lot of disposal alternatives that can be used under the same circumstances[90], and it is possible the QSTR model features do not sufficiently disclose the context of the moment of the decision. However, the convergence curve for other disposal QSTR models begins to trend downward after the seventh iteration and may continue even after the last iteration, as shown in Figure S2c. During the Bayesian optimization, the other disposal QSTR model improves by around 4% compared to the base model presented in Table S1. Despite the difficulty of convergence for the energy recovery and other disposal models, it is possible that these models may achieve accuracy greater than or close to 70% when evaluated on the test dataset.

S3.2.1. Multi-class classification

Figure S3 depicts the convergence curve for optimizing the multi-class classification QSTR model. The curve shows only the best value for the global function in an iteration. For example, at the first iteration, the best value for the objective function (i.e., - mean f1 score for 5-fold cross-validation) is -0.45. Figure S3 shows that the objective function moves from -0.45 to -0.47. The optimization trails reach a plateau around -0.47, i.e., after this value, the optimization cannot get a better score and the optimization is stopped after around 11 iterations with very small changes. The actual tuning time spent on optimizing the multi-class classification QSTR model is 12,954 seconds (including initial trials) with a minimum objective function value of -0.47. That means that the best mean f1 score achieved corresponds to 0.47, an improvement of only 4% with respect to the base QSTR model performance (see the bright red row in Table S2). Thus, it is not expected this model can achieve the desired performance in the external validation on the test dataset.

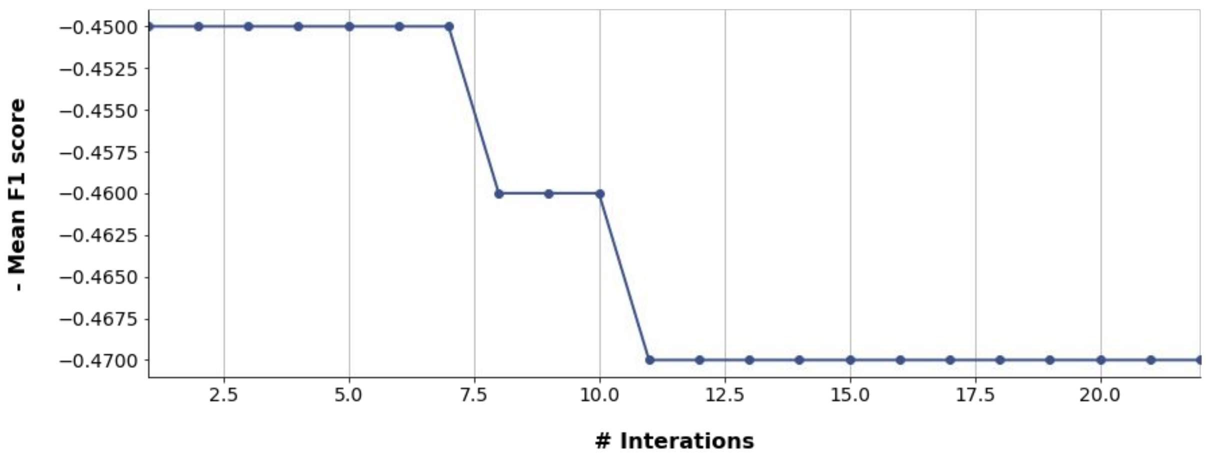

Figure S3. Convergence curve for the Bayesian optimization with Gaussian process performed for the QSTR model developed under the multi-class classification strategy.

S3.2.2. Multi-label classification

Figure S4 depicts the convergence curve for the hyperparameter optimization of the QSTR model built using a multi-label classification strategy. For the nine iterations presented in Figure S4, the best value reached by the objective function is -0.71. This value corresponds to the base QSTR model presented in the bright red row in Table S3. That means that no improvement is achieved after the Bayesian optimization, and because of the lack of a markable change in the convergence curve, the optimization is stopped. For this strategy, the actual tuning time spent is 12,832 seconds, including the time required for the random trials performed by the Bayesian optimization to obtain a first estimate of the surrogate function. Figure S4 indicates the difficulty of having a good performance for the model without any technique for solving a multi-label classification problem like problem transformation via label powerset or classifier chains [42]. Table S3 shows that the mean validation accuracy for the base QSTR model under this modeling strategy does not exceed 0.67. Therefore, this QSTR model is not expected to exceed the desired accuracy value of 70% for the test data set in the external validation.

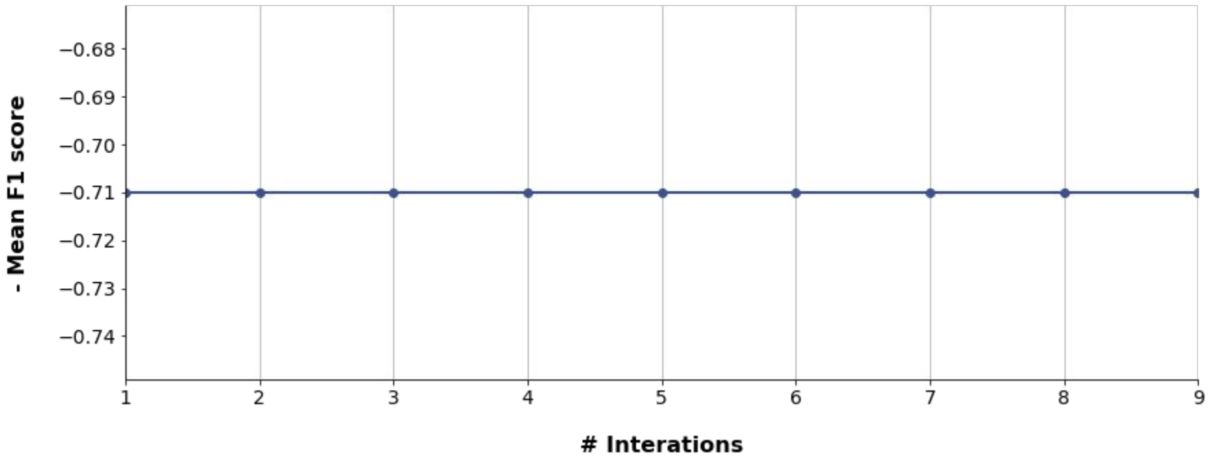

Figure S4. Convergence curve for the Bayesian optimization with Gaussian process performed for the QSTR model developed under the multi-label classification strategy.

S4. QSTR model deployment

The following link contains the GitHub repository with the scripts for the local deployment of the QSTR models:

https://github.com/jodhernandezbe/prtr_deployment

https://github.com/USEPA/PRTR-QSTR-models/tree/model-deployment

In addition, a RESTful API services deployed on the AWS contains the QSTR models. Someone can use the API for making prediction and embed the API as a micro-service into a personal project. Check the API documentation:

http://3.92.83.22:5000/v1/api_documentation

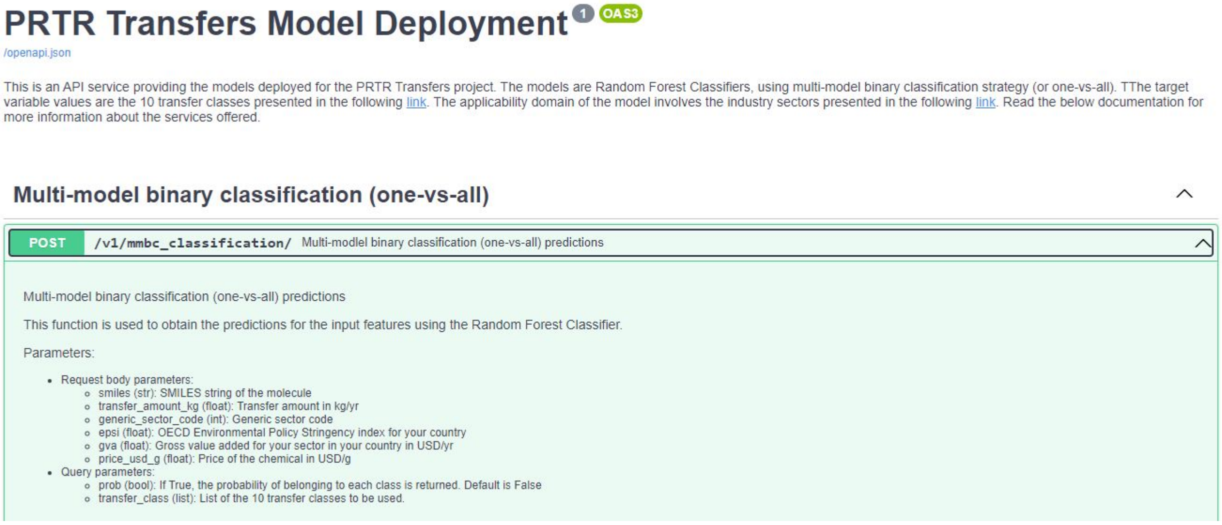

Figure S5. Screenshot of the documentation of the API developed for deploying the QSTR models

As the website uses a free dyno hour pool allocated by AWS, the API response can take a while.

References

[1] Patro, S. G. K.; Sahu, K. K. Normalization: A Preprocessing Stage. *IARJSET*, **2015**, 20–22. https://doi.org/10.17148/IARJSET.2015.2305.

[2] Singh, D.; Singh, B. Investigating the Impact of Data Normalization on Classification Performance. *Appl Soft Comput*, **2020**, *97*, 105524. https://doi.org/10.1016/j.asoc.2019.105524.

[3] Verma, R. P.; Hansch, C. An Approach toward the Problem of Outliers in QSAR. *Bioorg Med Chem*, **2005**, *13* (15), 4597–4621. https://doi.org/10.1016/j.bmc.2005.05.002.

[4] Zhang, K.; Hutter, M.; Jin, H. A New Local Distance-Based Outlier Detection Approach for Scattered Real-World Data; 2009; pp 813–822. https://doi.org/10.1007/978-3-642-01307-2_84.

[5] Liu, F. T.; Ting, K. M.; Zhou, Z.-H. Isolation Forest. In *2008 Eighth IEEE International Conference on Data Mining*; IEEE, 2008; pp 413–422. https://doi.org/10.1109/ICDM.2008.17.

[6] Hernandez-Betancur, J. D.; Ruiz-Mercado, G. J.; Martin, M. Tracking End-of-Life Stage of Chemicals: A Scalable Data-Centric Amd Chemical-Centric Approach (under Review). **2022**.

[7] LIU, H.; HUSSAIN, F.; TAN, C. L.; DASH, M. Discretization: An Enabling Technique. *Data Min Knowl Discov*, **2002**, *6*, 393–423. https://doi.org/https://doi.org/10.1023/A:1016304305535.

[8] SUN, Y.; WONG, A. K. C.; KAMEL, M. S. CLASSIFICATION OF IMBALANCED DATA: A REVIEW. *Intern J Pattern Recognit Artif Intell*, **2009**, *23* (04), 687–719. https://doi.org/10.1142/S0218001409007326.

[9] Chawla, N. V.; Bowyer, K. W.; Hall, L. O.; Kegelmeyer, W. P. SMOTE: Synthetic Minority Over-Sampling Technique. *Journal of Artificial Intelligence Research*, **2002**, *16*, 321–357. https://doi.org/10.1613/jair.953.

[10] Charte, F.; Rivera, A. J.; del Jesus, M. J.; Herrera, F. MLSMOTE: Approaching Imbalanced Multilabel Learning through Synthetic Instance Generation. *Knowl Based Syst*, **2015**, *89*, 385–397. https://doi.org/10.1016/j.knosys.2015.07.019.

[11] Zhang, J. P.; Mani, I. KNN Approach to Unbalanced Data Distributions: A Case Study Involving Information Extraction. In *Proceeding of International Conference on Machine Learning*; 2003.

[12] Johnstone, I. M.; Titterington, D. M. Statistical Challenges of High-Dimensional Data. *Philosophical Transactions of the Royal Society A: Mathematical, Physical and Engineering Sciences*, **2009**, *367* (1906), 4237–4253. https://doi.org/10.1098/rsta.2009.0159.

[13] Zong, P.; Jiang, J.; Qin, J. Study of High-Dimensional Data Analysis Based on Clustering Algorithm. In *2020 15th International Conference on Computer Science & Education (ICCSE)*; IEEE, 2020; pp 638–641. https://doi.org/10.1109/ICCSE49874.2020.9201656.

[14] Raj, E. *Engineering MLOps: Rapidly Build, Test, and Manage Production-Ready Machine Learning Life Cycles at Scale*, 1st ed.; Shaikh, N., Arjun, V., Eds.; Packt Publishing: Birmingham, 2021.

[15] Maaten, L. van der; Postma, E.; Herik, J. van den. Dimensionality Reduction: A Comparative Review. *JMLR, Oct 29*, **2009**, https://rb.gy/484st3 (accessed Nov 30, 2022).

[16] Kuhn, M.; Johnson, K. *Feature Engineering and Selection*, 1st ed.; Chapman and Hall, Ed.; Chapman and Hall/CRC: New York, 2019. https://doi.org/10.1201/9781315108230.

[17] Visbal-Cadavid, D.; Mendoza-Mendoza, A.; Hoz-Dominguez, E. D. La. Use of Factorial Analysis of Mixed Data (FAMD) and Hierarchical Cluster Analysis on Principal Component (HCPC) for Multivariate Analysis of Academic Performance of Industrial Engineering Programs. *Journal of Southwest Jiaotong University*, **2020**, *55* (5). https://doi.org/10.35741/issn.0258-2724.55.5.34.

[18] Potdar, K.; S., T.; D., C. A Comparative Study of Categorical Variable Encoding Techniques for Neural Network Classifiers. *Int J Comput Appl*, **2017**, *175* (4), 7–9. https://doi.org/10.5120/ijca2017915495.

[19] Polishchuk, P. G.; Muratov, E. N.; Artemenko, A. G.; Kolumbin, O. G.; Muratov, N. N.; Kuz'min, V. E. Application of Random Forest Approach to QSAR Prediction of Aquatic Toxicity. *J Chem Inf Model*, **2009**, *49* (11), 2481–2488. https://doi.org/10.1021/ci900203n.

[20] Svetnik, V.; Liaw, A.; Tong, C.; Culberson, J. C.; Sheridan, R. P.; Feuston, B. P. Random Forest: A Classification and Regression Tool for Compound Classification and QSAR Modeling. *J Chem Inf Comput Sci*, **2003**, *43* (6), 1947–1958. https://doi.org/10.1021/ci034160g.

[21] Biau, G. Analysis of a Random Forests Model. *J Mach Learn Res*, **2010**, *13*, 1063–1095.

[22] Sonar, R.; Deshmukh, P. R. Multiclass Classification: A Review. *International Journal of Computer Science and Mobile Computing*, **2014**, *3* (4), 65–69.

[23] Zhang, M.-L.; Zhou, Z.-H. A Review on Multi-Label Learning Algorithms. *IEEE Trans Knowl Data Eng*, **2014**, *26* (8), 1819–1837. https://doi.org/10.1109/TKDE.2013.39.

[24] Wu, X.; Gao, Y.; Jiao, D. Multi-Label Classification Based on Random Forest Algorithm for Non-Intrusive Load Monitoring System. *Processes*, **2019**, *7* (6), 337. https://doi.org/10.3390/pr7060337.

[25] Tsoumakas, G.; Katakis, I.; Vlahavas, I. Random K-Labelsets for Multilabel Classification. *IEEE Trans Knowl Data Eng*, **2011**, *23* (7), 1079–1089. https://doi.org/10.1109/TKDE.2010.164.

[26] Gramatica, P. Principles of QSAR Models Validation: Internal and External. *QSAR Comb Sci*, **2007**, *26* (5), 694–701. https://doi.org/10.1002/qsar.200610151.

[27] Bates, S.; Hastie, T.; Tibshirani, R. Cross-Validation: What Does It Estimate and How Well Does It Do It? *arXiv, Apr 1*, **2021**, https://rb.gy/wdbria (accessed Nov 30, 2022).

[28] Rücker, C.; Rücker, G.; Meringer, M. Y-Randomization and Its Variants in QSPR/QSAR. *J Chem Inf Model*, **2007**, *47* (6), 2345–2357. https://doi.org/10.1021/ci700157b.

[29] Ponzoni, I.; Sebastián-Pérez, V.; Martínez, M. J.; Roca, C.; De la Cruz Pérez, C.; Cravero, F.; Vazquez, G. E.; Páez, J. A.; Díaz, M. F.; Campillo, N. E. QSAR Classification Models for Predicting the Activity of Inhibitors of Beta-Secretase (BACE1) Associated with Alzheimer's Disease. *Sci Rep*, **2019**, *9* (1), 9102. https://doi.org/10.1038/s41598-019-45522-3.

[30] Wold, S.; Eriksson, L.; Clementi, S. Statistical Validation of QSAR Results; 1995; pp 309–338. https://doi.org/10.1002/9783527615452.ch5.

[31] Fawcett, T. An Introduction to ROC Analysis. *Pattern Recognit Lett*, **2006**, *27* (8), 861–874. https://doi.org/10.1016/j.patrec.2005.10.010.

[32] Sokolova, M.; Japkowicz, N.; Szpakowicz, S. Beyond Accuracy, F-Score and ROC: A Family of Discriminant Measures for Performance Evaluation; 2006; pp 1015–1021. https://doi.org/10.1007/11941439_114.

[33] Sorower, M. S. A Literature Survey on Algorithms for Multi-Label Learning. *Oregon State University, Dec*, **2010**, https://rb.gy/h7dbjf (accessed Nov 30, 2022).

[34] Sabaghi, M.; Mascle, C.; Baptiste, P.; Rostamzadeh, R. Sustainability Assessment Using Fuzzy-Inference Technique (SAFT): A Methodology toward Green Products. *Expert Syst Appl*, **2016**, *56*, 69–79. https://doi.org/10.1016/j.eswa.2016.02.038.

[35] Hernández-Betancur, J. D.; Hernández, H. F.; Ocampo-Carmona, L. M. A Holistic Framework for Assessing Hot-Dip Galvanizing Process Sustainability. *J Clean Prod*, **2019**, *206*, 755–766. https://doi.org/10.1016/j.jclepro.2018.09.177.

[36] Scheff, S. W. *Fundamental Statistical Principles for the Neurobiologist*; Elsevier, 2016. https://doi.org/10.1016/C2015-0-02471-6.

[37] Liashchynskyi, P.; Liashchynskyi, P. Grid Search, Random Search, Genetic Algorithm: A Big Comparison for NAS. *arXiv, Dec 12*, **2019**, https://rb.gy/ayr3ws (accessed Nov 30, 2022).

[38] Bergstra, J.; Bengio, Y. Random Search for Hyper-Parameter Optimization. *Journal of Machine Learning Research*, **2012**, *13*, 281–305.

[39] Brochu, E.; Cora, V. M.; de Freitas, N. A Tutorial on Bayesian Optimization of Expensive Cost Functions, with Application to Active User Modeling and Hierarchical Reinforcement Learning. *arXiv, Dec 12*, **2010**, https://rb.gy/8rnxim (accessed Nov 30, 2022).

[40] Rasmussen, C. E.; Williams, C. K. I. *Gaussian Processes for Machine Learning*; The MIT Press, 2006.

[41] Breiman, L. RANDOM FORESTS. *Mach Learn*, **2001**, *45*, 5–32. https://doi.org/10.1023/A:1010933404324.

[42] Pushpa, M.; Karpagavalli, S. Multi-Label Classification: Problem Transformation Methods in Tamil Phoneme Classification. *Procedia Comput Sci*, **2017**, *115*, 572–579. https://doi.org/10.1016/j.procs.2017.09.116.
